# Carcinome in situ conjonctival: une lésion rare à ne pas méconnaitre

**DOI:** 10.11604/pamj.2017.26.203.12075

**Published:** 2017-04-13

**Authors:** Rim Limaiem, Faten Limaiem

**Affiliations:** 1 Service d’Ophtalmologie B, Institut Hédi Rais d’Ophtalmologie, Tunisie; 2 Service d’Anatomie, Pathologique, Hôpital Mongi Slim La Marsa

**Keywords:** Néoplasie intra-épithéliale, conjonctive, carcinome in situ, œil, anatomie pathologique, Intraepithelial neoplasia, conjunctiva, eye, pathology

## Image en médecine

Un homme âgé de 30 ans sans antécédents pathologiques notables, avait consulté pour l'apparition trois mois auparavant d'une lésion cornéenne de l'œil droit. Dès l'examen par la simple inspection, une lésion saillante, accrochée au limbe temporal masquait la partie temporale de la cornée. Cette image se voyait confirmée par l'examen à la lampe à fente, la lésion était finement polylobée et avait un contour irrégulier vers la cornée. Elle empiétait à la fois sur la cornée et sur le versant limbique de la conjonctive (A). L'acuité visuelle était à 10/10. Le reste de l'examen ophtalmologique était normal. Devant cet aspect clinique évocateur d'une lésion tumorale de la surface oculaire droite, il fut proposé au patient une exérèse chirurgicale réglée. L'exérèse fut donc réalisée sous anesthésie locale sous microscope opératoire permettant une excision complète de la lésion. L'examen histologique a confirmé le diagnostic de carcinome in situ (B). Les suites opératoires immédiates étaient simples. Cependant, l'évolution a été marquée par la survenue six mois plus tard, d'une récidive locale imposant une réintervention chirurgicale.

**Figure 1:**
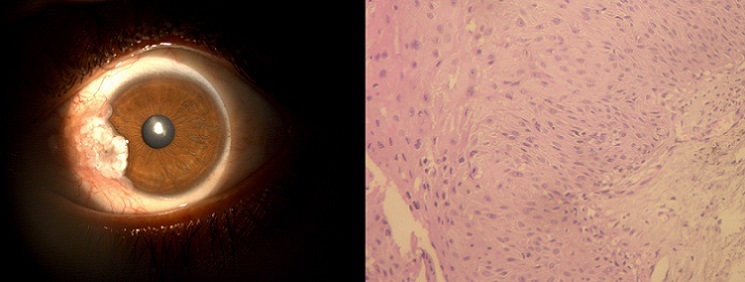
(A) l’examen à la lampe à fente, montrait une lésion blanchâtre finement polylobée à contours irréguliers vers la cornée. Elle empiétait à la fois sur la cornée et sur le versant limbique de la conjonctive; (B) Prolifération intra-épithéliale faite de cellules néoplasiques aux noyaux pléomorphes avec perte de la polarité. La lame basale était intacte dépourvue de signe d’envahissement tumoral (Hématoxyline et éosine, x 200)

